# Supplementary material for: An Accurate and Rapid Way for Identifying Food Geographical Origin and Authenticity: Editable DNA-Traceable Barcode
Source: Foods. 2022 Dec 21;12(1):17. doi: 10.3390/foods12010017 (PMC9818171; doi:10.3390/foods12010017)
Supplement: Supplementary file 1 [file foods-12-00017-s001.zip › foods-2067680-supplementary.pdf]

Table S1. The one-to-one mapping of writing information to nucleotides.

**Writing information**		**0**	**1**	**2**	**3**	**4**	**5**	**6**	**7**	**8**	**9**
**Nucleotides**	Index A	data	GAC	CAT	AAG	GGT	TAA	GTT	TAT	CCT	CTG
	Index B	data	GAT	AGC	CCT	ATC	GTC	ACC	CTC	AAG	AGT

File S1. The sequencing results for the original DNA-traceable barcode vector and the encapsulated and released DNA-traceable barcode vector are identical. The bolded part is the DNA-traceable barcode.

(1) Original DNA-traceable barcode vector sequence results

Primer M13-47

GGAAGAGGGTAGTGATTCGAGCTCGGTACCCGGGGATCCTCTAGAGATT**TGAGGCAAGG
ATTGAGGAAGATCGTCATACTTGTCATGATTTAGACCGGGACCGCCTCTGCGGAGGCT
AGAAGGAGAATAAAGGATGGGGGTCCTACCCCTCTTTCTACGCC**CAATCGTCGACCTG
CAGGCATGCAAGCTTGGCGTAATCATGGTCATAGCTGTTTCCTGTGTGAAATTGTTATCCG
CTCA

Primer RV-M

GACATTGACTGATTACGCCAGCTTGCATGCCTGCAGGTCGACGATT**GGCGTAGAAAGAG
GGGTAGGACCCCCATCCTTTATTCTCCTTCTAGCCTCCGCAGAGGCGGTCCCGGTCT
AAATCATGACAAGTATGACGATCTTCCTCAATCCTTGCCTCA**AATCTCTAGAGGATCCC
CGGGTACCGAGCTCGAATTCACTGGCCGTCGTTTTACAACGTCGTGACTGGGAAAACCCTT

(2) Encapsulated and released DNA-traceable barcode vector sequence results

Primer M13-47

CGTCGTGAATTCGAGCTCGGTACCCGGGGATCCTCTAGAGATT**TGAGGCAAGGATTGAG
GAAGATCGTCATACTTGTCATGATTTAGACCGGGACCGCCTCTGCGGAGGCTAGAAG
GAGAATAAAGGATGGGGGTCCTACCCCTCTTTCTACGCC**AATCGTCGACCTGCAGGCA
TGCAAGCTTGGCGTAATCATGGTCATAGCTGTTTCCTGTGTGAAATTTTATCCGCTCA

Primer RV-M

ACCTTGAACTGAT**TACGCC**AGCTTGCATGCCTGCAGGTCGACGATT**GGCGTAGAAAGAG
GGGTAGGACCCCCATCCTTTATTCTCCTTCTAGCCTCCGCAGAGGCGGTCCCGGTCT
AAATCATGACAAGTATGACGATCTTCCTCAATCCTTGCCTCA**AATCTCTAGAGGATCCC
CGGGTACCGAGCTCGAATTCACTGGCCGTCGTTTTACAACGTCGTGACTGGGAAAACCCTC

(3) Sequencing results of DNA-traceable barcode vector recovered from *Citrus sinensis* surface

Primer M13-47

GAGGGGTCCGTGATTCGAGCTCGGTACCCGGGGATCCTCTAGAGATT**TGAGGCAAGGAT
TGAGGAAGATCGTCATACTTGTCATGATTTAGACCGGGACCGCCTCTGCGGAGGCTA
GAAGGAGAATAAAGGATGGGGGTCCTACCCCTCTTTCTACGCC**AATCGTCGACCTGCA
GGCATGCAAGCTTGGCGTAATCATGGTCATAGCTGTTTCCTGTGTGAAATTTTATCCGCTC
A

Primer RV-M

CGGCACAAGCTTGCATGCCTGCAGGTCGACGATT**GGCGTAGAAAGAGGGGTAGGACCC
CCATCCTTTATTCTCCTTCTAGCCTCCGCAGAGGCGGTCCCGGTCTAAATCATGACA
AGTATGACGATCTTCCTCAATCCTTGCCTCA**AATCTCTAGAGGATCCCCGGGTACCGAG
CTCGAATTCACTGGCCGTCGTTTTACAACGTCGTGACTGGGAAAACCCTG
